# An administrative data validation study of the accuracy of algorithms for identifying rheumatoid arthritis: the influence of the reference standard on algorithm performance

**DOI:** 10.1186/1471-2474-15-216

**Published:** 2014-06-23

**Authors:** Jessica Widdifield, Claire Bombardier, Sasha Bernatsky, J Michael Paterson, Diane Green, Jacqueline Young, Noah Ivers, Debra A Butt, R Liisa Jaakkimainen, J Carter Thorne, Karen Tu

**Affiliations:** 1 University of Toronto, Toronto, 200 Elizabeth St 13EN-224, Toronto, ON M5G 2C4, Canada; 2 McGill University, Montreal, QC, Canada; 3 Institute for Clinical Evaluative Sciences, Toronto, ON, Canada; 4 McMaster University, Hamilton, ON, Canada; 5 Women’s College Hospital, Toronto, ON, Canada; 6 Sunnybrook Health Sciences Centre, Toronto, ON, Canada; 7 Southlake Regional Health Centre, Newmarket, ON, Canada

**Keywords:** Rheumatoid arthritis, Health administrative databases, Validation study, Sensitivity and specificity, Predictive values, Diagnostic test

## Abstract

**Background:**

We have previously validated administrative data algorithms to identify patients with rheumatoid arthritis (RA) using rheumatology clinic records as the reference standard. Here we reassessed the accuracy of the algorithms using primary care records as the reference standard.

**Methods:**

We performed a retrospective chart abstraction study using a random sample of 7500 adult patients under the care of 83 family physicians contributing to the Electronic Medical Record Administrative data Linked Database (EMRALD) in Ontario, Canada. Using physician-reported diagnoses as the reference standard, we computed and compared the sensitivity, specificity, and predictive values for over 100 administrative data algorithms for RA case ascertainment.

**Results:**

We identified 69 patients with RA for a lifetime RA prevalence of 0.9%. All algorithms had excellent specificity (>97%). However, sensitivity varied (75-90%) among physician billing algorithms. Despite the low prevalence of RA, most algorithms had adequate positive predictive value (PPV; 51-83%). The algorithm of “[1 hospitalization RA diagnosis code] or [3 physician RA diagnosis codes with ≥1 by a specialist over 2 years]” had a sensitivity of 78% (95% CI 69–88), specificity of 100% (95% CI 100–100), PPV of 78% (95% CI 69–88) and NPV of 100% (95% CI 100–100).

**Conclusions:**

Administrative data algorithms for detecting RA patients achieved a high degree of accuracy amongst the general population. However, results varied slightly from our previous report, which can be attributed to differences in the reference standards with respect to disease prevalence, spectrum of disease, and type of comparator group.

## Background

Validation of health administrative data algorithms for identifying patients with different health states is an important step in accurately using these data for secondary research [1]. Validation studies of administrative data algorithms often lack consistent methodology which can also make interpretation difficult [2]. For rheumatologic diseases of relatively low prevalence, researchers often sample patients from specialty clinics in order to increase the prevalence of case patients in their validation samples. However, this approach may elevate positive predictive values (PPV) and limit the generalizability of study results as not all patients receive continuous specialty-based care. In order to establish the optimal approach to identifying patients using administrative data, a population-based sample should be used, such that the disease prevalence in the validation cohort can approximate the prevalence of disease in the population [3,2].

Previously, we evaluated the accuracy of administrative data algorithms among a sample of 450 rheumatology clinic patients, in whom the prevalence of rheumatoid arthritis (RA) was 33%. We demonstrated that administrative data algorithms are highly accurate in identifying RA patients under active rheumatology care [4]. While these results are promising, it is not known whether the algorithms we tested perform equally well amongst the general population.

In Canada, primary care physicians act as ‘gate-keepers’ for access to specialists. Thus, primary care medical records represent a valuable resource for administrative data validation studies, as these records provide a closer approximation of population-based disease prevalence.

Thus, our primary objective was to reassess the accuracy of administrative data algorithms for identification of RA patients using diagnoses documented within the medical records of primary care physicians, and to compare our results with those of a previous study in which we used the medical records of rheumatologists as the reference standard [4].

## Methods

### Setting and design

In Ontario, all 13 million residents are covered by a universal, single-payer, public health insurance including hospital care and physicians’ services. A retrospective chart abstraction study was performed among a random sample of patients seen in primary care clinics to identify patients with and without RA. These patients were then linked to health administrative data to test and validate different combinations of physician billing, hospitalization and pharmacy data to identify RA patients within administrative data. This study was approved by the Sunnybrook Health Sciences Centre Research Ethics Board, and a waiver of patient consent was obtained as all analyses were performed on de-identified patient records.

### Participant selection

We used the Electronic Medical Record Administrative data Linked Database (EMRALD) [5]. Clinically relevant information contained in the patients’ charts includes all physician office visits, information on the patient’s current and past medical history, laboratory test results, prescriptions, specialist consultation letters, discharge summaries and diagnostic tests. We studied 83 physicians representing both urban and rural Ontario. A random sample of 7500 patients aged 20 years or older and 2,000 patients aged 65 years or older as of December 31, 2010 were drawn from 73,014 qualifying patients. Patients were included if they had a valid health insurance number and date of birth, had at least one visit in the previous year, and were rostered to (enrolled in) the physician’s practice. The latter criteria was required to ensure enough clinical information to verify disease status.

### Data abstraction

Using a standardized data abstraction tool, 9500 patients had their entire medical record screened by one of five trained abstractors to identify whether any had evidence of inflammatory arthritis. To assess the intra- and inter-rater reliability of chart abstractors, an initial 10% sample of charts was abstracted a second time by the same abstractor and once by a different abstractor. Kappa scores for inter- and intra-rater reliability exceeded 0.85 indicating good agreement for all five chart abstractors. Then, one abstractor (JW) reviewed the records of patients who, based on initial screen, were identified as possibly having inflammatory arthritis. The purpose of the second screen was to verify whether these patients had a diagnosis of RA, and whether a rheumatologist, orthopedic surgeon or internist had confirmed it. In addition, the abstractor verified drug history [i.e., prescription for non-steroidal anti-inflammatory drugs (NSAIDS), glucocorticosteroids, disease-modifying anti-rheumatic drugs (DMARDs), or biologics], and whether patients satisfied elements of RA classification criteria [6,7]. Results of serology and acute phase reactants tests were also obtained from both laboratory test fields within the electronic medical records (EMR) and specialist consultation notes. All trained chart abstractors were blinded to the administrative data diagnoses codes for all patients.

### Reference standard

Patients were classified as either RA cases or non-RA patients based on the level of evidence in the chart. The highest levels of evidence to support an RA diagnosis were: i) diagnosis by a rheumatologist, orthopedic surgeon, or internal medicine specialist; or ii) a primary care physician-documented RA diagnosis with supporting evidence (e.g., serology, joint involvement, or treatment) but without a supporting specialist consultation note. Additionally, patients were flagged as ‘possible RA’ cases if the record mentioned RA but lacked supporting evidence, or if the record had a “query RA” diagnosis. These ‘possible RA’ cases were used in a sensitivity analysis surrounding our reference standard definition of RA in which both definite RA and possible RA cases were grouped together.

### Health administrative data sources

Once patients were classified as having or not having RA according to our reference standard, administrative data were obtained for these patients for the period April 1, 1991 to March 31^st^ 2011 (the years for which administrative data were available during the study period). We used the Ontario Health Insurance Plan (OHIP) Database [8] to identify physician billing diagnosis codes. Physicians are reimbursed by submitting claims to OHIP for medical services provided. One diagnosis code, representing the main ‘reason for the visit’, is provided with each claim. These diagnoses are coded according to a modification of the 8th revision of the International Classification of Diseases (ICD) [9]. Hospital visits were identified using the Canadian Institute for Health Information Discharge Abstract Database (CIHI-DAD), which contains detailed information regarding all hospital admissions, and the National Ambulatory Care Reporting System (NACRS), which records all hospital-based and community-based ambulatory care for day surgery and emergency rooms (ERs) [10]. Hospital data prior to 2002 have diagnoses coded in ICD-9 [9] and can contain up to 16 diagnoses recorded per hospital encounter. Hospitalizations and ER encounters after 2002 are coded using ICD-10, and each record contains up to 25 diagnoses per encounter. For patients aged 65 years or older, medication exposures for DMARDS, biologics, and glucocorticosteroids, were determined using the pharmacy claims database of the Ontario Drug Benefit (ODB) Program [11]. Information on physician specialty for the billing claims was obtained by linking the Institute for Clinical Evaluative Sciences Physician Database (IPDB) with the OHIP database [12]. Musculoskeletal specialists were identified as rheumatologists, orthopedic surgeons, and internal medicine specialists. The OHIP Registered Persons Database (RPDB) contains a single unique record for each health care beneficiary that enables the linkage of these datasets in an anonymous fashion using encrypted health insurance numbers [13]. All data reside and all analyses were performed at the Institute for Clinical Evaluative Sciences (http://www.ices.on.ca).

### Test methods

Results are reported using the modified Standards for Reporting of Diagnostic Accuracy (STARD) criteria [14,3]. Our methodological approach and study conduct complies with recent recommendations on the reporting and study design of administrative data validation studies [2].

### Algorithm testing

Algorithms were derived using combinations of physician billing diagnosis (ICD 714), primary and secondary hospital discharge diagnosis (ICD9 714; ICD10 M05-M06), prescription drug claims (for DMARDs, biologics, and glucorticosteroids), by varying windows between diagnosis codes or the period in which diagnosis codes appeared, and whether the services were rendered by a musculoskeletal specialist. Additionally, we tested algorithms with exclusion criteria in which diagnosis codes for ‘other rheumatology conditions’ appeared after a diagnosis of RA, or for which an RA diagnosis code was not provided when a patient was seen by a rheumatologist. The ‘other rheumatology diagnoses’ included: osteoarthritis, gout, polymyalgia rheumatica, other seronegative spondyloarthropathy, ankylosing spondylitis, connective tissue disorder, psoriasis, synovitis/tenosynovitis/bursitis, and vasculitis. All algorithms were tested on the entire sample except those involving prescription drug claims, which were limited to patients aged 65 years and older. The algorithms were applied to all administrative data for the study period (up to March 31, 2011).

### Statistical analysis

Descriptive statistics were used to characterize the study population. We computed the sensitivity, specificity, positive predictive value (PPV) and negative predictive value (NPV) of each algorithm with corresponding 95% confidence intervals (CI). Details of the how these measures were computed are illustrated in Table 1[2]. Algorithms were first ranked according to highest PPV, followed by highest sensitivity and specificity. All analyses were performed on de-identified data using SAS version 9.2 (SAS Institute, Cary, North Carolina).

**Table 1:** Methods to computing measures of diagnostic accuracy

		**Reference standard**		$\text{Pre}-\text{test}\;\text{prevalence}=\frac{\text{TP}\;+\;\text{FN}}{\text{TP}\;+\;\text{FP}\;+\;\text{FN}\;+\;\text{TN}}\;$
		**RA cases**	**Non-cases**	
Administrative data	Positive	True Positive (TP)	False Positive (FP)	$\text{PPV}=\frac{\text{TP}}{\text{TP}+\text{FP}}$
	Negative	False Negative (FN)	True Negative (TN)	$\text{NPV}=\frac{\text{TN}}{\text{FN}\;+\;\text{TN}}$
		$\text{Sensitivity}=\frac{\text{TP}}{\text{TP}\;+\;\text{FN}}$	$\text{Specificity}\;=\;\frac{\text{TN}}{\text{FP}\;+\;\text{TN}}$	$\text{Post}-\text{test}\;\text{Prevalence}=\frac{\text{TP}\;+\;\text{FP}}{\text{TP}\;+\;\text{FP}\;+\;\text{FN}\;+\;\text{TN}}$

### Sample size

We estimated that we would require at least 50 individuals with RA in order to obtain sensitivity and specificity estimates of approximately 85% with a precision that ensured that the 95% CIs were within +/− 5% of the estimate. Assuming an overall RA prevalence of 0.7-1% in the adult population, we required a study cohort comprising at least 7000 adult patients. Taking into account that RA prevalence increases in a mature population (>65 years of age) [15], we estimated that we would need a random sample of approximately 2000 additional seniors in order to have enough RA cases aged >65 to ensure precise results when we restrict our analysis to seniors and incorporate drug prescriptions in our administrative algorithm. Therefore, stratified random sampling was performed to obtain 7500 patients aged 20 years and older for our primary analysis and add an additional 2000 patients aged 65 and older for our analyses restricted to seniors.

## Results

**Figure 1:**
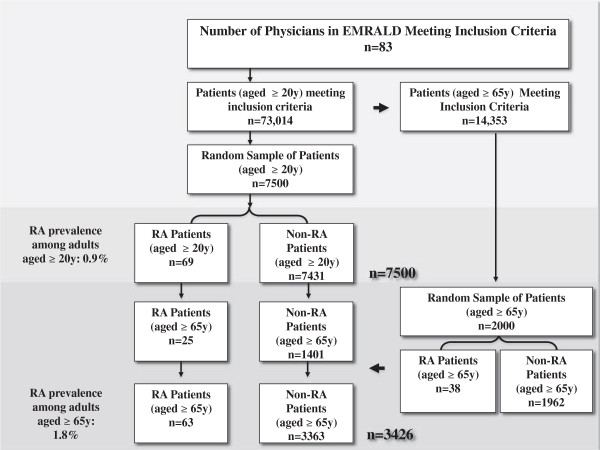
Flow diagram of selection of study participants.

The 83 physicians included in this study were in practice an average of 18.4 years, and they had used an EMR an average of 5.9 years (standard deviation, SD 3.2). The average patient time on the EMR was 5 years (range 2–23.5). Analyses were performed on 7500 patients aged 20 years and older and 3426 patients aged 65 years and older. The average age of the 7500 patients in the adult cohort was 49 (SD 17, range: 20–98) years.Using our reference standard definition, we identified 69 RA patients for a lifetime RA prevalence of 0.9% for patients aged 20 years and older (Figure 1). Amongst the RA cases, almost two thirds were female (64%) and the average age was 61.9 (SD 13.7) years. Amongst the 3426 patients aged 65 years and older, 63 (1.8%) patients had RA. Sixty-five percent were female and their average age was 76.3 (SD 6.8) years.

Clinical characteristics of the 69 RA patients are shown in Table 2. Most RA patients (86%) had documentation of an RA diagnosis by a specialist. Most RA patients also had documentation of bilateral joint involvement and small joint synovitis. Seropositivity (for rheumatoid factor and/or anti-ccp) was documented for 39% of RA patients and elevated acute phase reactants (i.e. erythrocyte sedimentation rate or c-reactive protein) were documented for 45%. DMARDs were the most commonly reported RA drug exposures and were present in 80% of patients.

**Table 2:** Clinical characteristics and drug exposures for RA patients

**Characteristics**	**Aged 20 and older n = 69**
Age (years), mean (SD)	61.9 (13.7)
Female gender	64%
Diagnosis provided by a Specialist	86%
Morning Stiffness	65%
Hand joint involvement (>1 swollen wrist, MCP, or PIP joint)	67%
Symmetric Arthritis (PIPs, MCPs, or MTPs)	65%
Rheumatoid Nodules	19%
Radiographic changes typical of RA	22%
2-10 medium-large joint involvement (shoulders, elbows, hips, knees, ankles)	39%
1-3 small joint involvement (MCPs, PIPs, 2^nd^-5^th^ MTPs, thumb IPs, wrists)	54%
4-10 small joint involvement (as above)	51%
>10 joint involvement (as above)	26%
RF or ACPA positive	39%
Elevated ESR or CRP	45%
NSAID/COXIB use*	77%
Glucocorticosteroid use (Oral, inter-articular or intramuscular)*	61%
DMARD use*	80%
Biologic use*	17%

*Prescribed by any physician (specialist or primary care physician).

*Abbreviations*: *MCPs* metacarpophalangeal joints, *PIPs* proximal interphalangeal joints, *MTPs* metatarsophalangeal joints, *IPs* interphalangeal joints, *DMARD* disease-modifying anti-rheumatic drug, *NSAID* non-steroidal anti-inflammatory drug, *COXIB* COX-2 selective inhibitor, *RF* Rheumatoid Factor, *ACPA* anti–citrullinated protein antibody, *ESR* erythrocyte sedimentation rate, *CRP* C-reactive protein.

Sensitivity, specificity, and predictive values for selected administrative data algorithms are reported in Table 3. Algorithms selected for reporting are based on those commonly in use by researchers. Access to the complete set of over 100 additional algorithms is available through the corresponding author. All algorithms had excellent specificity (97-100%). However, the sensitivity of the physician billing algorithms varied (77-90%). Despite the low prevalence of RA in the primary care records, algorithms for identifying RA patients had at least modest PPV (51-83%), which improved substantially with the requirement for musculoskeletal specialist billings. Varying the duration of the observation window for diagnosis codes had little impact on the accuracy of the algorithms. The addition of time restrictions between diagnosis codes (e.g., diagnosis codes ≥ 8 weeks apart) and exclusion criteria also did not improve algorithm performance.

**Table 3:** Test characteristics of multiple algorithms among patients ≥ 20 y

**Algorithms [Pretest prevalence: 0.9%]**	**TP**	**TN**	**FN**	**FP**	**Post-test prev.**^**#**^	**Sensitivity [95 CI%]**	**Specificity [95 CI%]**	**PPV [95 CI%]**	**NPV [95 CI%]**
1 H ever	15	7429	54	2	0.2%	22 (12–32)	100 (100–100)	88 (73–100)	99 (99–100)
1 H ever OR 1 ER ever	16	7427	53	4	0.3%	23 (13–33)	100 (100–100)	80 (63–98)	99 (99–100)
1 P ever	62	7188	7	243	4.1%	90 (83–97)	97 (96–97)	20 (16–25)	100 (100–100)
1 P ever by a specialist	56	7377	13	54	1.5%	81 (72–90)	99 (99–100)	51 (42–60)	100 (100–100)
2 P by any physician in 1 YR	58	7363	11	68	1.7%	84 (75–93)	99 (99–99)	46 (37–55)	100 (100–100)
2 P by any physician in 2 YR	58	7359	11	72	1.7%	84 (75–93)	99 (99–99)	45 (36–53)	100 (100–100)
2 P by any physician in 3 YR	58	7352	11	79	1.8%	84 (75–93)	99 (99–99)	42 (34–51)	100 (100–100)
3 P by any physician in 1 YR	55	7398	14	33	1.2%	80 (70–89)	100 (99–100)	63 (52–73)	100 (100–100)
3 P by any physician in 2 YR	55	7395	14	54	1.2%	80 (70–89)	100 (99–100)	60 (50–71)	100 (100–100)
3 P by any physician in 3 YR	55	7393	14	38	1.2%	80 (70–89)	100 (99–100)	59 (49–69)	100 (100–100)
2 P with ≥ 1 P by a specialist in 1 YR	54	7404	15	27	1.1%	78 (69–88)	100 (100–100)	67 (56–77)	100 (100–100)
2 P with ≥ 1 P by a specialist in 2 YR	54	7404	15	27	1.1%	78 (69–88)	100 (100–100)	67 (56–77)	100 (100–100)
2 P with ≥ 1 P by a specialist in 3 YR	54	7401	15	30	1.1%	78 (69–88)	100 (100–100)	64 (54–75)	100 (100–100)
3 P with ≥ 1 P by a specialist in 1 YR	53	7419	16	12	0.9%	77 (67–87)	100 (100–100)	82 (72–91)	100 (100–100)
3 P with ≥ 1 P by a specialist in 2 YR	53	7418	16	13	0.9%	77 (67–87)	100 (100–100)	80 (71–90)	100 (100–100)
3 P with ≥ 1 P by a specialist in 3 YR	53	7418	16	13	0.9%	77 (67–87)	100 (100–100)	80 (71–90)	100 (100–100)
(1 H ever) OR (2 P with ≥ 1 P by a specialist in 1 YR)	55	7403	14	28	1.1%	80 (70–89)	100 (100–100)	66 (56–76)	100 (100–100)
(1 H ever) OR (2 P with ≥ 1 P by a specialist in 2 YR)	55	7403	14	28	1.1%	80 (70–89)	100 (100–100)	66 (56–76)	100 (100–100)
(1 H ever) OR (2 P with ≥ 1 P by a specialist in 3 YR)	55	7400	14	31	1.1%	80 (70–89)	100 (99–100)	64 (54–74)	100 (100–100)
(1 H ever) OR (3 P with ≥1 P by a specialist in 1 YR)	54	7417	15	14	0.9%	78 (69–88)	100 (100–100)	79 (70–89)	100 (100–100)
(1 H ever) OR (3 P with ≥1 P by a specialist in 2 YR)	54	7416	15	15	0.9%	78 (69–88)	100 (100–100)	78 (69–88)	100 (100–100)
(1 H ever) OR (3 P with ≥1 P by a specialist in 3 YR)	54	7416	15	15	0.9%	78 (69–88)	100 (100–100)	78 (69–88)	100 (100–100)
(1 H ever) OR (2 P ≥ 8 weeks apart in 2 YR, no exclusions*	57	7378	12	53	1.5%	83 (74–92)	99 (99–100)	52 (43–61)	100 (100–100)
(1 H ever) OR (2 P ≥ 8 weeks apart in 2 YR) excluding Case A or Case B	41	7401	28	30	0.9%	59 (48–71)	100 (100–100)	58 (46–69)	100 (100–100)
(1 H ever) OR (2 P ≥ 8 weeks apart in 3 YR) excluding Case A or Case B	41	7396	28	35	1.0%	59 (48–71)	100 (99–100)	54 (43–65)	100 (100–100)
(1 H ever) OR (2 P ≥ 8 weeks apart in 4 YR) excluding Case A or Case B	43	7394	26	37	1.1%	62 (51–74)	100 (99–100)	54 (43–65)	100 (100–100)
(1 H ever) OR (2 P ≥ 8 weeks apart in 5 YR) excluding Case A or Case B	44	7390	25	41	1.1%	64 (52–75)	99 (99–100)	52 (41–62)	100 (100–100)
(1 H ever) OR (2 P ≥ 8 weeks apart in 2 YR) excluding Case A	41	7402	28	29	0.9%	59 (48–71)	100 (100–100)	59 (47–70)	100 (100–100)

All Ages: N = 7500 patients (69 RA and 7431 non-RA); ^#^ = Post-test prevalence; TP = True Positive; TN = True Negative; FN = False Negative; FP = False Positive

H: Hospitalization diagnosis code; P = physician diagnosis code; Specialist = rheumatologist, internal medicine, orthopedic surgeon; *Exclusions: Case A: Patients with ≥ 2 P with another rheumatology diagnosis subsequent to an RA diagnosis OR Case B: Patients for which the diagnosis of RA was not confirmed by a specialist. Other rheumatology diagnoses include: Osteoarthritis (715), Gout (274, 712), Polymyalgia Rheumatica (725), other seronegative spondyloarthropathy (721), Ankylosing spondylitis (720), psoriasis (696), synovitis/tenosynovitis/bursitis (727), connective tissue disorder (710), vasculitis (446), others (716; 718; 728; 727; 728; 729; 781).

Among seniors (Table 4), the requirement for an RA drug exposure slightly improved the PPV, although the 95% CIs overlapped with those for the algorithms excluding RA drugs.

**Table 4:** Test characteristics of multiple algorithms among patients aged ≥ 65 y

**Algorithms [Pretest prevalence: 1.8%]**	**TP**	**TN**	**FN**	**FP**	**Post-test prev.**^**#**^	**Sensitivity [95 CI%]**	**Specificity [95 CI%]**	**PPV [95 CI%]**	**NPV [95 CI%]**
1 P AND ≥1 Rx ever	53	3248	10	115	4.9%	84 (75–93)	97 (96–97)	32 (25–39)	100 (100–100)
2 P AND ≥1 Rx ever	51	3318	12	45	2.8%	81 (71–91)	99 (98–99)	53 (43–63)	100 (99–100)
2 P ≥ 60 days apart AND ≥1 RX ever	49	3320	14	43	2.7%	78 (68–88)	99 (98–99)	53 (43–64)	100 (99–100)
(1 H ever) OR (2 P AND ≥1 Rx in 1 YR)	52	3337	11	26	2.3%	83 (73–92)	99 (99–100)	67 (56–77)	100 (100–100)
(1 H ever) OR (2 P AND ≥1 Rx in 2 YR)	52	3335	11	28	2.3%	83 (73–92)	99 (99–100)	65 (55–76)	100 (100–100)
(1 H ever) OR (2 P AND ≥1 RX in 3 YR)	52	3332	11	31	2.4%	83 (73–92)	99 (99–99)	63 (52–73)	100 (100–100)
(1 H ever) OR (2 P with ≥ 1 P by specialist AND ≥1 Rx in 1 YR)	52	3347	11	16	2.0%	83 (73–92)	100 (99–100)	77 (66–87)	100 (100–100)
(1 H ever) OR (2 P with ≥ 1 P by specialist AND ≥1 Rx in 2 YR)	52	3346	11	17	2.0%	83 (73–92)	100 (99–100)	75 (65–86)	100 (100–100)
(1 H ever) OR (2 P with ≥ 1 P by specialist AND ≥ 1 Rx in 3 YR)	52	3345	11	18	2.0%	83 (73–92)	100 (99–100)	74 (64–85)	100 (100–100)
(1 H ever) OR (3 P AND ≥ 1 Rx in 1 YR)	47	3346	16	17	1.9%	75 (64–85)	100 (99–100)	73 (63–84)	100 (99–100)
(1 H ever) OR (3 P AND ≥ 1 Rx in 2 YR)	48	3342	15	21	2.0%	76 (66–87)	99 (99–100)	70 (59–80)	100 (99–100)
(1 H ever) OR (3 P AND ≥ 1 Rx in 3 YR)	48	3342	15	21	2.0%	76 (66–87)	99 (99–100)	70 (59–80)	100 (99–100)
(1 H ever) OR (3 P with ≥ 1 P by specialist AND ≥ 1 Rx in 1 YR)	47	3351	16	12	1.7%	75 (64–85)	100 (99–100)	80 (69–90)	100 (99–100)
(1 H ever) OR (3 P with ≥ 1 P by specialist AND ≥ 1 Rx in 2 YR)	48	3349	15	14	1.8%	76 (66–87)	100 (99–100)	77 (67–88)	100 (99–100)
(1 H ever) OR (3 P with ≥ 1 P by specialist AND ≥ 1 Rx in 3 YR)	48	3348	15	15	1.8%	76 (66–87)	100 (99–100)	76 (66–87)	100 (99–100)

Seniors 65+: N = 3426 patients (63 RA and 3363 non-RA); ^#^ = Post-test prevalence; TP = True Positive; TN = True Negative; FN = False Negative; FP = False Positive; H: Hospitalization code; P = physician diagnosis code; Specialist = rheumatologist, internal medicine, orthopedic surgeon; Rx refers to drug exposures: oral corticosteroid, disease-modifying anti-rheumatic drug (DMARD) or biologic; DMARDs included: Azathioprine, Chloroquine, Cyclophosphamide, Cyclosporine, Gold, Minocycline; and Biologics included: Etanercept, Adalimumab, Infliximab, Certolizumab, Golimumab; Abatacept, Anakinra, Rituximab, Tocilizumab.

After ranking algorithms by our a priori definition, the algorithm to meet this criteria was: “[1 hospitalization RA code] OR [3 physician RA diagnosis codes (claims) with ≥1 by a specialist in a 2 year period]” which had a sensitivity of 78% (95% CI 69–88), specificity of 100% (95% CI 100–100), PPV of 78% (95% CI 69–88) and NPV of 100% (95% CI 100–100).

When we varied the definition of our reference standard to include all possible RA patients, the prevalence increased from 0.9% to 1.3%. When the algorithms were retested including these patients, there was a trend toward decreasing sensitivity. There was no effect on specificity or NPV, however PPV slightly increased with more RA patients classified in the reference standard.

## Discussion

Our findings show that, in the primary care setting, most administrative data algorithms for RA had high specificity. We found that incorporating specialist diagnosis codes increased PPV (51-83%), and requiring multiple RA codes increased both specificity and PPV. However increasing the duration of the observation window to identify RA codes or varying the time between RA codes had little impact on algorithm performance. In addition, incorporating RA drugs only slightly improved PPV, hospitalization codes alone had poor sensitivity, and use of more complex cases definitions involving exclusion criteria did not improve algorithm performance.

Overall, we have comprehensively evaluated the accuracy of administrative data algorithms that were measured relative to the reference standards of two independent validation samples using the diagnoses documented within medical charts of rheumatologists (previously reported) [4] and family physicians (reported here). After testing administrative data algorithms and ranking algorithms according to performance characteristics, the optimal algorithm identified for identifying RA patients in both samples was identical. The algorithm of “[1 hospitalization RA code] OR [3 physician RA diagnosis codes with ≥1 RA code by a specialist in a 2 year period]” had a sensitivity of 78%, specificity of 100%, PPV of 78% and NPV of 100% when using our primary care reference standard. When we independently validated this algorithm among a random sample of 450 patients seen in rheumatology clinics [4], it demonstrated a sensitivity of 97%, specificity of 85%, PPV of 76% and NPV of 98%.

While we identified the same algorithm as optimal in both settings, we did not achieve identical results, which can be attributed to differences in the study samples (reference standards) with respect to disease prevalence, spectrum of disease, and type of comparator group. For example, 33% of patients had RA within the rheumatology sample [4] versus 0.9% within the primary care sample. The spectrum of disease (clinical characteristics) in our rheumatology sample included contemporary RA patients under active rheumatology care and treatment, compared to patients with a lifetime RA diagnosis who may only be currently receiving active primary care. The type of comparator group (non-cases) in our rheumatology study involved patients with other rheumatologic diagnoses, in contrast to our primary care patients who are healthy or have other diagnoses.

In both samples, algorithm sensitivity was computed only among study subjects with RA, and specificity was computed among those without RA. Sensitivity and specificity do not depend on the prevalence of the RA in the study population, but they can vary across populations [16]. For example, sensitivity or our administrative data algorithm was excellent (>97%) at identifying contemporary RA under active rheumatology care [4]. In contrast, sensitivity was moderately good (78%) at identifying RA patients with a lifetime diagnosis of RA (who include patients under active rheumatology care, but also patients whose symptoms may have resolved, and are no longer seeking RA care). When we varied our definition of RA based on levels of evidence in the primary care charts (i.e., varied the spectrum of disease in our cohort), more strict definitions of RA according to the reference standard increased sensitivity, whereas more liberal definitions of RA (such as allowing any mention of RA with no supporting evidence, or those with a query RA diagnosis) decreased sensitivity of the administrative data algorithms. Thus, defining an a priori reference standard to classify individuals with RA has implications for validation study methodology. For instance, patients who fulfill strict classification criteria, such as the 1987 RA criteria [17], may have more advanced disease (patients with longer disease duration, or more active disease requiring multiple physician visits) and have a greater chance of being detected by administrative data algorithms. This finding was also observed in a review of studies that tested administrative data algorithms amongst RA patients who were required to meet strict classification criteria: these patients had a higher sensitivity in comparison to patients who were classified by more liberal criteria (such as an RA diagnosis documented in the medical record) [2].

On the other hand, as specificity is computed only among those without RA, the type of patients included in the non-RA comparator group (as defined by the reference standard) can influence the estimates of specificity. The lower specificity observed amongst non-RA patients in the rheumatology clinic study (85% versus 100% specificity observed amongst the primary care sample), is reflective of the patients without RA in the rheumatology clinic sample are individuals with other rheumatologic diagnoses (which may resemble RA, and/or who at one point in time may have been considered a possible RA patient, and then evolved into a more clear diagnosis, such as systemic lupus). In contrast, the comparator group of individuals without RA in our primary care sample included patients with other conditions (unrelated to RA), which improved the specificity. This observation has implications for research that seeks to identify population-based algorithms with high specificity. Furthermore, it emphasizes the importance of reporting the characteristics of the patients in the comparator group to inform proper interpretation of specificity estimates.

While sensitivity and specificity are dependent on the characteristics of patients with and without the disease, respectively, predictive values depend on disease prevalence, in addition to sensitivity and specificity. An important finding of our study is that our optimal algorithm had virtually the same PPV (78%) in both studies. Our primary care sample had an RA prevalence reflective of that of the general population (0.9%) in comparison to the rheumatology sample which had a study RA prevalence of 33% [4]. However, there was variation among the PPV estimates ranging from 51-83% amongst our primary care sample, and ranging from 55-80% amongst the rheumatology sample. The PPV estimates improved substantially with the requirement of increasing the number of diagnosis codes and including musculoskeletal specialist codes for RA. In addition to differences in prevalence estimates in both samples, specificity differed, with all algorithms tested within our primary care sample achieving very high (≥97%) specificity. In general, for conditions that are present in a minority of the study population (such as our primary care sample), specificity has a greater impact than sensitivity on PPV. However specificity alone is not the sole factor increasing PPV amongst the primary care sample, as all algorithms with high specificity would have moderately good PPV. A more likely explanation is that the preferred algorithm identified fewer false positives in both settings, as having fewer false positives increases PPV. This is likely owing to the nature of RA management, which often involves referral to a specialist, frequent physician visits and a non-curative long disease course. These observations suggest that patients with prevalent (long-term) chronic conditions may have a higher probability of being identified by the use of similar administrative data algorithms owing to the disease course, management practices, and frequency of physician visits. This finding may be further supported by the concordance between our results and those from algorithms for case ascertainment of diabetes [17], hypertension [18], and other chronic diseases with substantially higher prevalence than RA.

This study has both strengths and limitations. Patients were randomly sampled from primary care physician records and we tested many more permutations of administrative data algorithms than other studies. We also conducted rigorous chart reviews. However, misclassification of RA is a potential risk if there is lack of documentation in the medical record, such as a failure to capture all specialist consultation notes. Further, clinical characteristics for the RA patients may be under-documented in primary care clinical records. Recognizing this challenge, we opted to include all physician-reported diagnoses to define our reference standard, as our retrospective study design can make it difficult to determine true disease status. However, our present findings extend those of previous research performed using the General Practice Research Database, which also found a sensitivity of 80% for patients with 3 RA diagnosis codes [19]. Further, as the purpose of our study was to test the accuracy of algorithms for classifying RA patients within administrative data, we were unable to confirm the validity of the diagnosis of RA itself (whether doctors were correctly diagnosing RA). However, the majority of RA patients had specialist notes to confirm RA.

Another potential limitation is that our findings are derived from patients who have a regular source of primary care. Consequently, our results may not be generalizable to patients who do not have a regular primary care physician. Although almost ten million Ontarians (that is, over 80% of the population) are now rostered to a primary care physician [20,21], we acknowledge this limitation and opted to include inpatient RA diagnosis codes in our final preferred administrative algorithm, even though alone these codes had low sensitivity (22%) and offered little improvement over physician-billing algorithms. The addition of an inpatient RA code to “3 physician RA claims with ≥1 by a specialist in a 2 year period” may subsequently increase the sensitivity of our algorithm when it is applied to the entire population since hospitalization data may be needed to pick up RA cases who either have no regular physician, or who are followed by the approximately 5% of Ontario physicians who are salaried (and who do not necessarily contribute to billing data) [22].

In addition, while the overall goal was to recommend the optimal algorithm for use in Ontario, we report the results of numerous algorithms so that researchers can be better informed by choosing the case definition best suitable for their study populations *and* study purpose. Due to different characteristics inherit to different administrative databases it would be imprudent to suggest preferred algorithms for use outside of Canada, where other researchers may be better informed on the characteristics of their own databases under study. Rather, algorithms should be selected based on study purpose and feasibility and weigh the relative importance of accuracy measures that is most important to a particular study. Incorrectly choosing the wrong algorithm or prioritizing the wrong accuracy measure can lead to misclassification, which can lead to reduced power, loss of generalizability, as well as increased bias, and possibly study cost [23].

## Conclusions

Our study demonstrates that administrative data algorithms can detect RA patients who receive regular primary care with a high degree of accuracy. However, accuracy varied slightly with the use of an alternative reference standard. Our findings will inform future population-based research and will serve to improve arthritis research surveillance activities across Canada and abroad.

## Competing interests

The authors declare that they have no competing interests related to this manuscript. Financial support provided by the Canadian Institutes of Health Research (operating grant 119348). This study was also supported by ICES, a non-profit research corporation funded by the Ontario Ministry of Health and Long-Term Care (MOHLTC). The opinions, results and conclusions are those of the authors and are independent from the funding sources. No endorsement by ICES or the Ontario MOHLTC is intended or should be inferred. We thank Brogan Inc., Ottawa for use of their Drug Product and Therapeutic Class Database.

## Authors’ contributions

JW, CB, SB, JMP, NI, DAB, RLJ, JCT and KT participated in the design of the study. JW, DG and JY performed the statistical analysis. All authors participated in interpreting results, drafting the manuscript and approved the final manuscript.

## Pre-publication history

The pre-publication history for this paper can be accessed here:

http://www.biomedcentral.com/1471-2474/15/216/prepub
